# Relative blood loss in forensic medicine—do we need a change in doctrine?

**DOI:** 10.1007/s00414-020-02260-w

**Published:** 2020-03-06

**Authors:** Stefan Potente, Frank Ramsthaler, Mattias Kettner, Patrick Sauer, Peter Schmidt

**Affiliations:** 1 Department of Legal Medicine, University of Saarland, Geb. 49.1, Kirrberger Strasse, 66421, Homburg, Germany; 2 Department of Legal Medicine, Goethe-University Frankfurt am Main, Kennedyallee 104, 60596, Frankfurt am Main, Germany

**Keywords:** Exsanguination, Reference values, Blood loss, Forensic, Haemorrhagic shock, Hypovolemic shock, ATLSⓇ

## Abstract

In forensic medicine, blood loss is encountered frequently, either as a cause of death or as a contributing factor. Here, risk to life and lethality assessment is based on the concept of *relative* blood loss (absolute loss out of total volume). In emergency medicine, the Advanced Trauma Life Support (ATLSⓇ) classification also refers to relative blood loss. We tested the validity of relative blood loss benchmarks with reference to lethality. Depending on the quality of the total blood volume (TBV) estimation formula, relative blood loss rates should be reflected in the case cohort as significantly higher absolute blood loss in heavier individuals since all TBV estimation formulas positively correlate body weight with TBV. Method: 80 autopsy cases with sudden, quantifiable, exclusively internal blood loss were retrospectively analyzed and a total of 8 different formulas for TBV estimation were applied. Results: No statistical correlation between body weight and absolute blood loss was found for any of the tested TBV estimation algorithms. All cases showed a wide spread of both absolute and relative blood loss. Discussion: The principle of relative blood loss is of very limited use in casework. It opens the forensic expert opinion to unnecessary criticism and possible negative legal implications. Conclusion: We challenge the use of relative blood loss benchmarks in textbooks and practical casework and advocate for its elimination from the ATLSⓇ ’s grading system. If necessary, we recommend the use of BMI-adjusted algorithms for TBV estimation.

## Introduction

### Lethality and relative blood loss in forensic medicine

Blood loss is a frequent finding in routine forensic pathology case work. It may constitute a genuine cause of death (in 5 to 10% of forensic autopsies, according to [1]) or a contributing factor tovadjust another primary cause of death. Forensic pathology doctrine, as referenced in different textbooks, relates lethality and risk of death to the *relative blood loss* (blood lost out of total blood volume). A relative blood loss of more than 33% $\left (\frac {1}{3}\right )$ has been described as life-threatening [2, 3], whereas 50% was seen as ‘usually lethal’. Other authors have described relative blood loss percentages for acute blood loss. Here, a loss of 40% and ‘more than 66%’$\left (\frac {2}{3}\right )$ have been categorized as ‘lethal’ [4, 5].

In addition to absolute and relative loss, internal blood loss with collection of blood in cavities and defined spaces may cause compressive mechanical effects, such as in the skull, the pericardial sac or the thoracic cavity [6].

### The Advanced Trauma Life Support (ATLSⓇ) classification of hypovolemic shock

A basic understanding of blood loss assessment in emergency medicine and pre-clinical trauma scenarios is needed in any assessment of forensic cases.

In primary clinical care, the Advanced Trauma Life Support (ATLS®) classification of hypovolemic shock is used. Patients suffering from blood loss are classified depending on heart rate, systolic pressure, pulse pressure and other parameters. Relative blood loss is part of the classification system, where 7% of body weight is assumed for TBV [7].

- Class I haemorrhage constitutes up to 15% blood loss, equivalent to an individual who has donated a unit of blood.
- Class II haemorrhage constitutes 15 to 30% blood loss, uncomplicated haemorrhage, a ‘mildly anxious’ patient and a simple crystalloid fluid replacement therapy.
- Class III haemorrhage constitutes 30 to 40% blood loss, a complicated haemorrhage, described as ‘devastating’, an ‘anxious and confused’ patient and imperative crystalloid infusions, possibly blood infusions needed.
- Class IV haemorrhage constitutes > 40% blood loss and is considered a preterminal event. Without very aggressive measures, the patient is expected to die within minutes.

The ATLSⓇ-classification has been criticized as a mere teaching tool rather than a practical guideline, as the postulated classes are rarely found in real-life scenarios [8].

### Autopsy findings

**Fig. 1:**
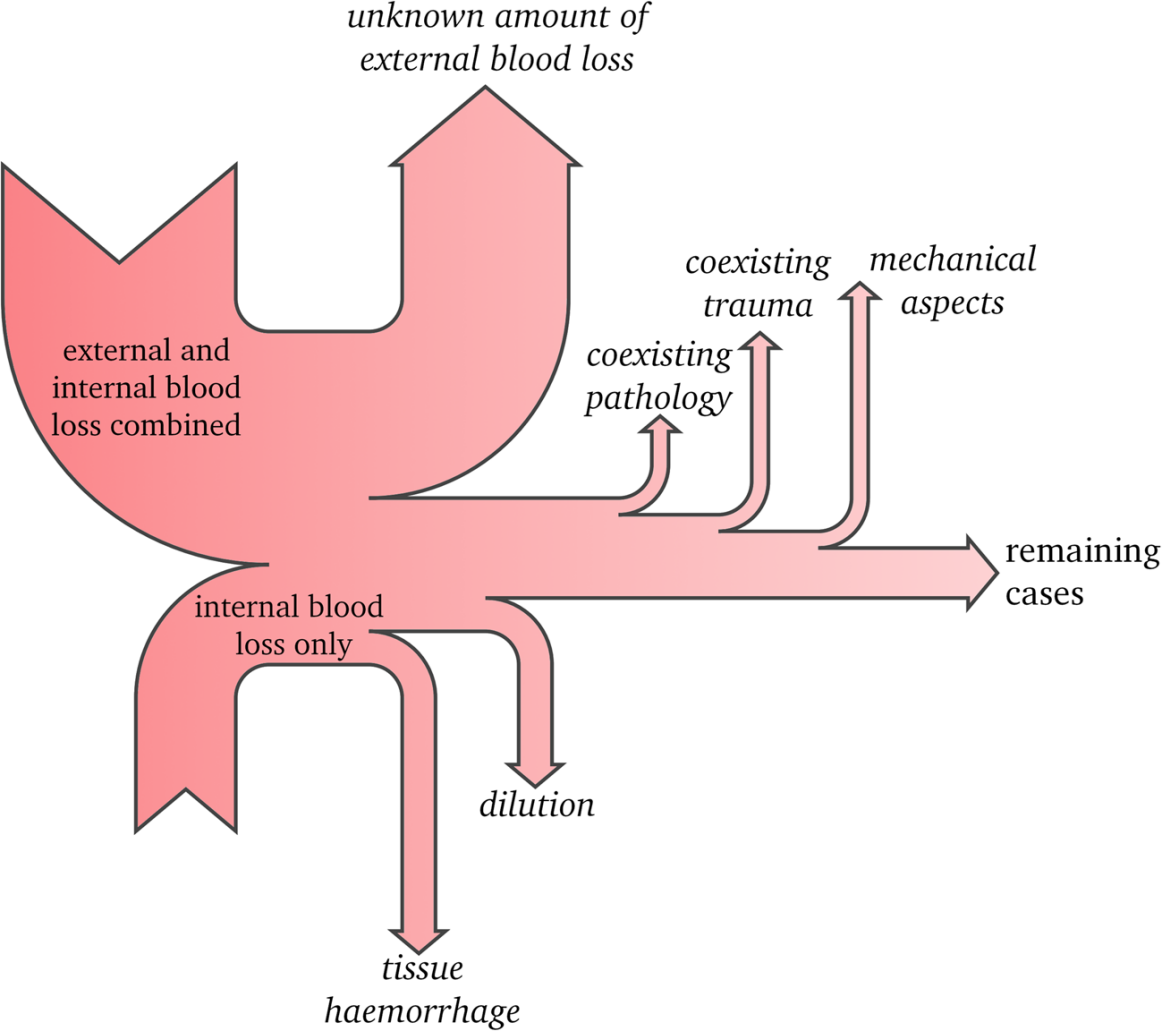
Pitfalls in the general assessment of relative blood loss during autopsy (Sankey style diagram)

The estimation of blood loss is part of routine autopsy in which free blood quantities are collected from the body cavities and measured. A variety of factors negatively influence the assessment of relative blood loss during autopsy (see Fig. 1). In many forensic cases, blood loss is both internal and external, with external blood loss rarely being precisely quantifiable. Internal blood loss is difficult to assess in cases of diffuse tissue haemorrhage and dilution with for example edema or intestinal contents. Also, the extent of blood loss may be limited by coexisting internal diseases and/or coexisting trauma leading to ‘premature’ decompensation as well as mechanical aspects of the bleeding itself such as in pericardial tamponade, intracranial haemorrhage and structural damage to the heart. Cardiopulmonary resuscitation (CPR) may prolong the extravasation of blood after cessation of circulation. Also, injuries as complications of resuscitation attempts, sometimes dramatic, have been reported frequently (for example [9, 10]). Classical autopsy findings of blood loss, besides a secured source of bleeding and possible pooling of blood, include sparse lividity, organ pallor, subendocardial haemorrhage, wrinkling of the spleen capsule and ‘shock kidneys’. They are however not present in every case.

### Rules of thumb and dedicated formulas for total blood volume (TBV) estimation

TBV is often generalized as 5 to 6 l for most individuals. In addition, there are a variety of rules of thumb and dedicated formulas for TBV estimation. More recent body-mass-index(BMI)-adapted formulas have, to our knowledge, not been used in legal medicine. Rules of thumb for the estimation of TBV, as published in forensic textbooks and scientific articles, are simple correlations of TBV with body weight, either as ‘volume per body weight’ (‘reference book method’, ‘index based’), a percentage of body weight or a baseline volume combined with a percentage of body weight (see Table 1). For these rules of thumb, we could not find scientific evidence, and more variations may exist.

**Table 1:** Rules of thumb for the estimation of total blood volume

Rule of thumb, principles	Examples of formula variations
Volume per body weight,	♂: TBV = 70 to 80 cm^3^/kg body weight [2]
‘reference book method’,	♀: TBV = 60 to 70 cm^3^/kg body weight [2]
‘blood volume index based’	
	♂: TBV = 70 ml/kg body weight [17]
	♀: TBV = 65 ml/kg body weight [17]
Baseline volume and percentage	♂: TBV = 7.0 L ± 0.5% body weight [4]
	♀: TBV = 6.5 L ± 0.5% body weight [4]
	♂: TBV = 0.041⋅kg+ 1.53 [3]
	♀: TBV = 0.047⋅kg+ 0.86 [3]
Percentages of body weight	TBV = 8% of body weight [18]
	♂: TBV = 7.5% of body weight [19]
	♀: TBV= 7.0% of body weight [19]

Cited are textbooks and scientific articles using or recommending the respective rule

Formulas listed in Table 2 use non-linear BMI-/constitution-dependent scaling. Non-linear TBV dependency on weight and constitution has been acknowledged in clinical medicine for some time [11–13]. The developed algorithms have been used clinically, especially in obesity surgery, but have not yet been used in the context of forensic pathology. Overweight persons constitute a significant proportion of the general population as well as forensic autopsy cases. For example, Sheikhazadi et al. [14] reported a mean BMI of 25.12 for autopsies in Tehran (*n*= 1222).

**Table 2:** BMI-/constitution- dependent formulas for TBV estimation

Author(s)	Formula
Nadler, Hidalgo, Bloch [20]	♂: TBV = (0.3669 ⋅ m^3^) + (0.03219 ⋅ kg) + 0.6041
	♀: TBV = (0.3561 ⋅ m^3^) + (0.03308 ⋅ kg) + 0.1833
Lemmens, Bernstein, Brodsky [21]	${\text {TBV=kg}} \cdot \frac {\text {70}}{\sqrt {\frac {\text {BMI}}{\text {22}}}}$
	with
	$\text {BMI=}\frac {\text {kg}}{\text {m}^{2}}$
friesen [22, 23] using	♀: 14148/(8780 + 244⋅ BMI)=LSF
Janmahasatian’s [24] lean-scaled	♂: 11432/(6680 + 216 ⋅ BMI)=LSF
factor (LSF)	Lean-scaled weight = body weight ⋅ LSF
	♀: TBV = 60 ml/kg lean-scaled weight
	♂: TBV = 70 ml/kg lean-scaled weight
Gilcher’s Rule of Five [25]	‘Normal’ body - ♂: TBV = 70 cm^3^/kg body weight,
	♀: TBV = 65 cm^3^/kg body weight
	‘Thin’ body - ♂: TBV = 65 cm^3^/kg body weight,
	♀: TBV = 60 cm^3^/kg body weight
	‘Obese’ body - ♂: TBV = 60 cm^3^/kg body weight,
	♀: TBV = 55 cm^3^/kg body weight
	‘Muscular’ body - ♂: TBV = 75 cm^3^/kg body weight,
	♀: TBV = 70 cm^3^/kg body weight

Original source cited. Note that there are metric and imperial measurement formulas available for Nadler et al. Table shows metric formula

Remarkably, in allometry, blood volume in interspecific comparison is treated as constant and no power-laws apply: TBV = 70 ml/kg body weight [15] or TBV ≈ 6–7% of body volume (for all mammals except aquatic ones) [16].

## Method

### Retrospective analysis

Autopsy reports from the forensic medicine departments of the University of Saarland Medical School in Homburg/Saar and the Goethe University Frankfurt Medical School in Frankfurt/Main were retrospectively screened for ‘bleeding to death’, exsanguination, haemorrhagic shock and hypovolemic shock as cause of death. In some cases, classification as primarily due to exsanguination was retrospectively rejected. During restrospective analysis of autopsy reports, the cohort had to be restricted to cases of sudden, quantifiable and exclusively internal haemorrhage due to difficulties described in ‘Autopsy findings’. The inclusion criteria were defined as sudden, quantifiable (minimum 600 ml) blood loss into the thoracic cavities and/or the abdominal cavity. Following this procedure, 80 cases remained. For these, amount, source and location of internal blood loss were noted as well as sex, age, height, weight, case history including resuscitation efforts, and cause of death. In total, eight formulas were applied to each case:

- Two percentage-based formulas: ‘P7’ and ‘P8’ (7% and 8% of body weight)
- One index-based formula: ‘Index’ (60 ml/kg (♀), 70 ml/kg (♂))
- Two baseline formulas: ‘BL1’ (6.5 L (♀) / 7.0 L (♂) ± 0.5% body weight) and ‘BL2’ (0.041 ⋅ kg + 1.53 (♂), 0.047 ⋅ kg + 0.86 (♀))
- Three BMI-dependent formulas (see Table 2): ‘NHB’ (Nadler, Hidalgo and Bloch), ‘LBB’ (Lemmens, Bernstein and Brodsky) and ‘FR’ (friesen)

In addition, the autopsy reports were screened for the findings listed below. The gradual transition of findings could not be systematically reflected in this process, due to the differences in description by various examiners in the retrospective setting:

- Reduced or absent lividity (either named as such or described as reduced in extent or confluence),
- Organ pallor (either named as such or described as pale, light, ‘of inherent color’ and the like),
- Subendocardial haemorrhage (either present or not),
- Shock kidneys (either named as such or described as pronounced demarcation between pale kidney cortex and dark medulla),
- Wrinkling of spleen capsule (either present or not).

In addition to descriptive statistics, confirmative analysis was performed using pearson’s correlation coefficient *r* with *p* value to analyze the degree of association between two or more variables as well as Levene’s test for comparing CPR-cases with non-CPR-cases (Python library Pandas [26]). Visualization was performed using the Python libraries Seaborn and Matplotlib [27].

### Nomographs

**Fig. 2:**
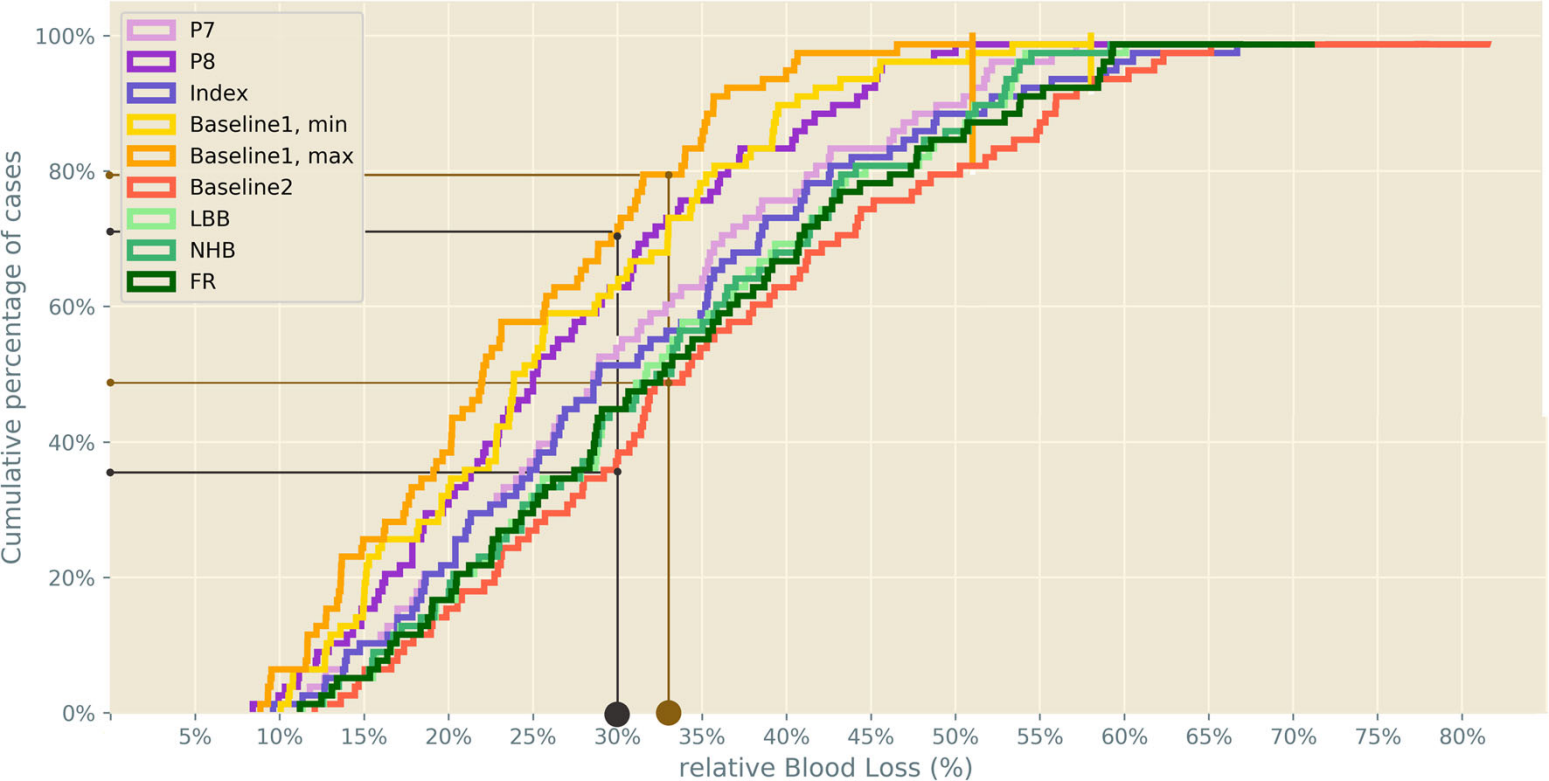
Nomographic representation of Lemmens, Bernstein and Brodsky’s formula for TBV estimation: connect body height (m) on the left scale with body weight (kg) on the right, then read TBV (l) on center scale

**Fig. 3:**
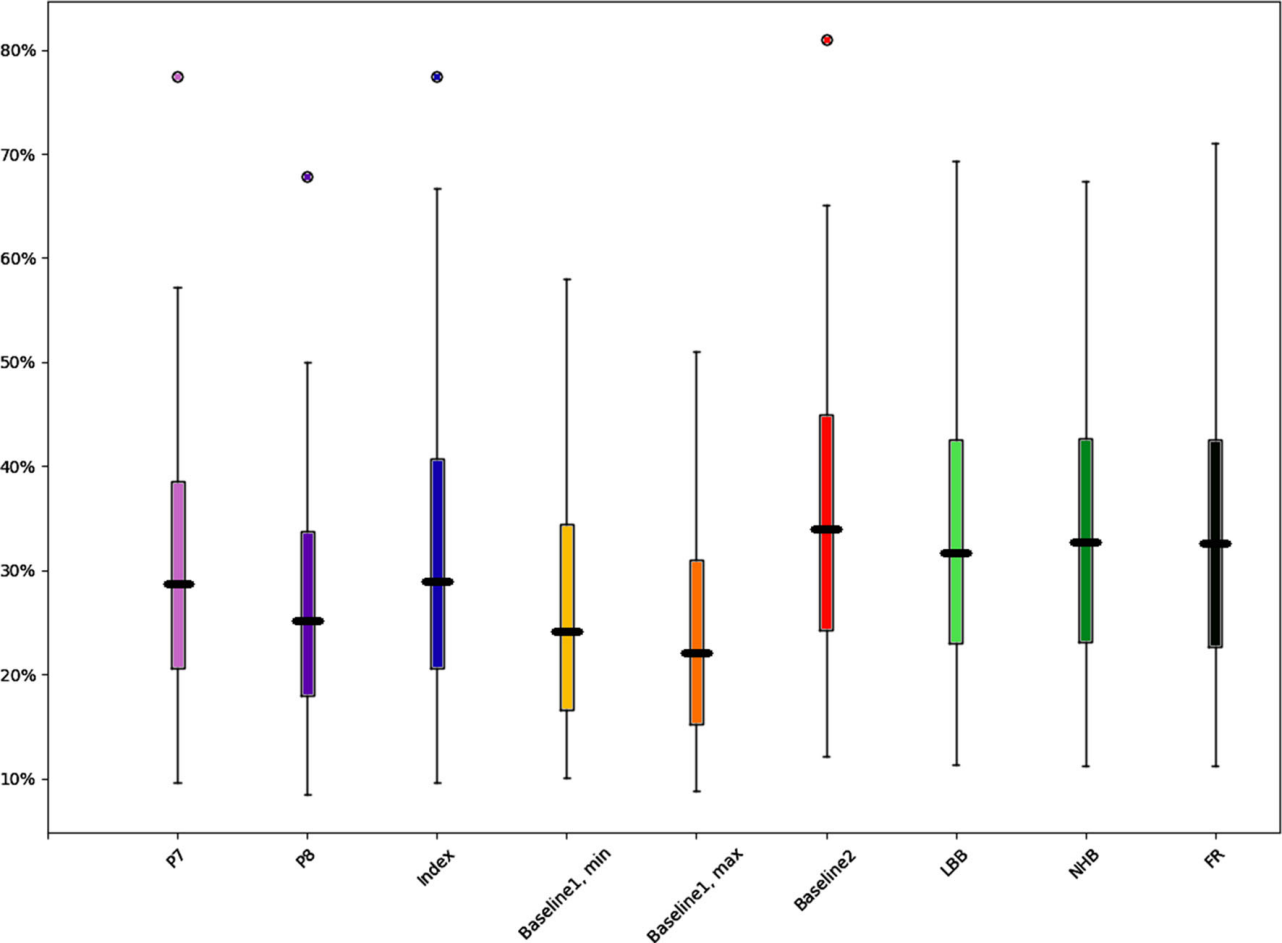
Nomographic representation of Nadler’s formula for TBV estimation: connect body weight (kg) on the left scale with body height (m) on bottom scale (read female on upper part, male on lower part of scale). Then, read TBV (l) on diagonal scale

Nomographs for the BMI-dependent algorithms by Lemmens et al. (see Fig. 2) and nadler et al. (see Fig. 3) were designed using the PyNomo [28] package for Python. The approach by friesen produces results close to Lemmens et al. and was therefore omitted.

## Results

### Absolute and relative blood loss for a variety of formulas and algorithms

The absolute blood loss found at autopsy ranged from 660 ml (chosen cut-off value 600 ml) up to 3800 ml (2 outliers, see ‘Discussion’).

In comparison, the *calculated* TBV using the 8 formulas ranged from 2280 ml (♀, 38 kg, 1.46 m, BMI 17.8, 31 years), calculated using the Index-formula (60 ml/kg, ♀), to 16,080 ml (♂, 201 kg, 1.75 m, BMI 65.6, 57 years) using the 8%-formula.

There was no statistically significant correlation between either age and total blood loss, weight and total blood loss or BMI and total blood loss. In particular, older age was not correlated with less blood loss, no higher susceptibility to blood loss could be found. Heavier persons did not lose higher amounts of blood. BMI-adapted formulas produced more moderate TBV estimations for overweight and obese individuals, apart from that none of the algorithms and formulas was particularly outstanding in terms of indication of a life-threatening condition. The amount of blood loss as well as relative blood loss (based on TBV estimation using various formulas) was seemingly randomly distributed across cases of all ages and body constitutions.

Relative blood loss of 30% (ATLSⓇ Class III, complicated haemorrhage) was not reached between 37% (Baseline2) and 72% of cases (Baseline1, maximum TBV) with average 53% of cases for all 8 algorithms.

**Fig. 4:**
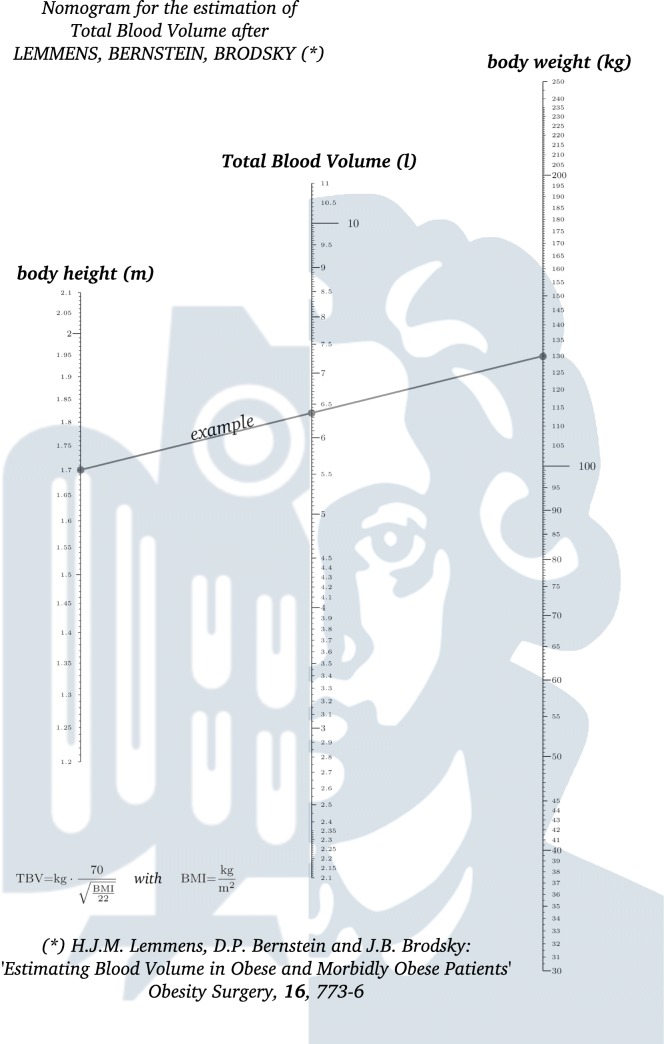
Cumulative percentages of relative blood loss (%) for various formulas and algorithms (*n*= 78). Grey: 30% threshold (ATLSⓇ). Brown: $\frac {1}{3}$ threshold (legal medicine doctrine)

Relative blood loss of 33% (one-third being described as ‘life-threatening’ in forensic literature) was not reached between 49% (Baseline2) and 79% of cases (Baseline 1, maximum TBV) with average 61% of cases for all 8 algorithms. A maximum of 20% of cases lost > 50% of their TBV when using the Baseline2 formula (see Fig. 4).

**Fig. 5:**
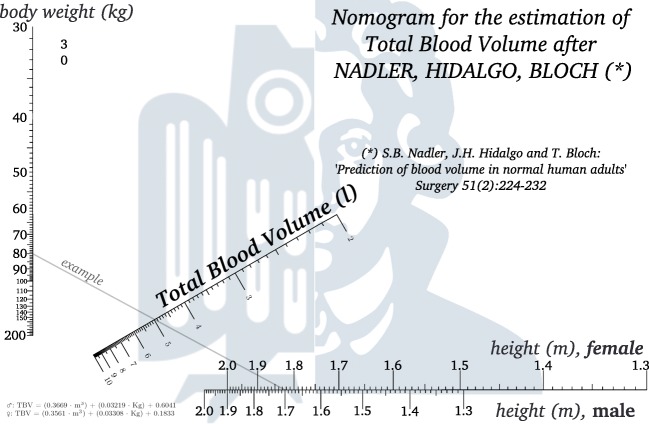
Calculated relative blood loss rates for various formulas and algorithms (*n*= 78)

For 8 algorithms, 9 TBV estimations were performed, as Baseline1 formula differs for minimum and maximum estimations. The lowest relative blood loss was calculated as only 8.4% (P8), the highest as 81.0% (Baseline2). The mean relative blood loss ranged from 23.6% (Baseline1, maximum) to 35.9% (Baseline 2). The overall mean relative blood loss for all cases and algorithms was 30.7% (see Fig. 5).

### Autopsy findings

Overall, the most frequent finding was organ pallor (80.7%), followed by shock kidneys (62.8%), subendocardial haemorrhage (58.9%), wrinkling of the spleen capsule (57.6%) and reduced lividity (51.2%).

However, only 4 cases (5.1%) showed a combination of all five findings, followed by two findings in 35.9%, three findings in 21.8%, four findings in 17.9% and one finding in 15.4 %.

For 3 cases (3.8%) none of the typical autopsy findings was described, despite individual absolute blood losses of 2100 ml (2 cases) and 2500 ml. All 3 cases involved rupture of the aorta due to internal causes.

Due to the inclusion criteria, the sources for bleeding cannot be generalized. In our limited cohort we found:

- Dissections and ruptures of the thoracic aorta,
- Trauma of the heart and lungs both isolated and combined,
- Combined lacerations of thoracic organs and crushing of ribs and tissues,
- Liver punctures,
- Ruptured aneurysms of the abdominal aorta,
- Others, including vena cava, esophagus, punctured kidney.

Trauma originated mainly from either deceleration injury in motor vehicle accidents or crushing of some sort. Due to the inclusion criteria of quantifiable/quantified blood loss volume, retroperitoneal haemorrhage following ruptured aneurysm of the abdominal aorta was underrepresented, since retroperitoneal blood accumulation was often not quantified absolutely but rather described or measured two dimensionally only.

### Cardiopulmonary resuscitation, CPR

For 66 out of 78 cases, it could be established whether CPR had been performed (*n*= 40) or not (*n*= 26). While cases with CPR had a higher mean absolute blood loss than cases without CPR (1.797 ml versus 1540 ml), this difference was not significant (levene’s test *p*= 0.22). In one case, all internal blood loss detected at autopsy could be directly attributed to chest compression during CPR (see below).

### Special cases

Two cases stood out for which according to most TBV estimation formulas, the absolute amount of blood loss *exceeded* the estimated TBV. In one case, a total of 5350 ml was present in the thoracic cavities. Evaluation of clinical documents showed that the patient had received blood transfusions which had cumulated in the chest cavities. The second case showed a total of 4500 ml blood in the abdominal cavity. On critical examination however, the deceased had severe liver cirrhosis which not only contributed to the extensive blood loss from spleen rupture due to falling but also had already produced ascitic fluid before the trauma, which had mixed with blood. Both cases were excluded from analysis.

In one additional case, all blood loss present at autopsy could be attributed to CPR alone: a 73-year-old female patient (165 cm, 75 kg) suffered complications in the form of intraabdominal haemorrhage during gall bladder surgery. The bleeding was stopped, blood products were administered and the operation was concluded. After extubation and relocation to the recovery room however, the patient suffered cardiac arrest and died despite immediate CPR. Autopsy revealed multiple rib fractures due to CPR with 2 fragments protruding into the thoracic cavity. Both the left cardiac atrium and the right ventricle were ruptured as well as the pericardial sac with a total of 1500 ml of blood in the left thoracic cavity.

## Discussion

The concept of relative blood loss can be utilized for only a small proportion of autopsy cases with significant blood loss. In the larger proportion of cases, the absolute blood loss cannot be determined precisely, mainly due to external blood loss but also because of diffuse haemorrhage into soft tissues, the gastrointestinal tract or dilution with edematous and other fluids. It is often assumed that bleeding from smaller and/or less pressurized vessels can be sustained for longer and damage to large vessels in close proximity to the heart, such as the aorta, may lead to loss of pressure and decompensation with relatively less blood loss. In those cases where absolute blood loss could be quantified, there is often significant collateral trauma and/or internal disease present. In these cases, circulatory decompensation as a limiting factor for an otherwise more extensive haemorrhage can hardly be ruled out. Nevertheless, these cases were included into our analysis and only cases of unknown absolute blood loss were excluded.

The concept of relative blood loss is a direct and plausible consequence of the facts that small amounts of blood loss are harmless (therefore ‘greater’ loss is needed to harm) and that total blood volume depends on body weight. However, relative blood loss as part of forensic pathology doctrine as well as ATLSⓇ classes performs poorly when applied to autopsy cases. Expected correlations, such as increased absolute blood loss for increased body weight, as well as varying resilience to blood loss for different age groups, have not materialized. Around half of the cases do not reach calculated relative blood loss rates as presented for threat to life in legal medicine doctrine and the ATLSⓇ classification. This may leave forensic expert opinions vulnerable to unnecessary criticism even in obvious cases:

Consider a case where an obese person (105 kg, 176 cm, BMI= 33.9) is killed with a gunshot through the aorta. 1.5 liters of blood are recovered from the left thoracic cavity. By all calculations, the threshold for ‘threat to life’ is not reached. It could be argued that a collateral pathology was likely present that was not foreseeable and without which the victim would have survived.

Should the question of total blood volume arise in any legal context, we advise to use the algorithms by Lemmens/Bernstein/Brodsky or Nadler/Hidalgo/ Bloch, since for overweight and obese individuals, overestimation of TBV is otherwise likely. Also, the algorithms are comparatively well documented. In addition, we have included nomographs for both, which can be used quickly with one stroke of a pen. After completion, the nomographs can be added to the case file.

## Conclusion

In ATLSⓇ, relative blood loss, presented as a percentage of TBV, may be removed from the classification system for hypovolemic shock without negative consequences.

In legal medicine, the concept can only be exerted on a fraction of all cases, when all limitations are accounted for. It constitutes an oversimplified rule of thumb and is poorly documented. It is therefore not suitable as a generalized concept in legal medicine and should be either amended or removed completely from textbooks. For practical casework, we advise to describe haemorrhage in broader terms (such as ‘light, moderate, severe’), refer to *absolute* rather than relative blood loss (‘... an amount which is found in comparable cases’) and to discuss the possible role of collateral trauma and pathology specific to the case. When applicable, underlying trauma or ‘polytrauma’ (as defined in [29]) should be emphasized as cause of death without explicit reference to any non-negligible volume of blood loss.
